# Current Transition Practice for Primary Immunodeficiencies and Autoinflammatory Diseases in Europe: a RITA-ERN Survey

**DOI:** 10.1007/s10875-022-01345-y

**Published:** 2022-10-12

**Authors:** Muskan Israni, Bethany Nicholson, Nizar Mahlaoui, Laura Obici, Linda Rossi-Semerano, Helen Lachmann, Georgia Hayward, Mojca Zajc Avramovič, Aurelien Guffroy, Virgil Dalm, Rachel Rimmer, Leire Solis, Carlotta Villar, Andrew R. Gennery, Stephanie Skeffington, Julia Nordin, Klaus Warnatz, Anne-Sophie Korganow, Jordi Antón, Marco Cattalini, Tania Amin, Stephan Berg, Pere Soler-Palacin, Siobhan O. Burns, Mari Campbell, RITA-ERN Transition Working Group Consortium, C. Wouters, I. Meyts, J. E. van der Werff ten Bosch, L. Goffin, B. Ogunjimi, O. Gilliaux, J. Kelecic, M. Jelusic, Š. Fingerhutová, A. Sediva, T. Herlin, R. J. Seppänen Mikko, K. Aalto, H. Ritterbusch, A. Insalaco, V. Moschese, A. Plebani, R. Cimaz, C. Canessa, R. M. Dellepiane, M. Carrabba, F. Barzaghi, J. A. M. van Laar, N. M. Wulffraat, L. Marques, C. Carreras, J. Sánchez-Manubens, L. Alsina, M. E. Seoane Reula, A. Mendez-Echevarria, L. I. Gonzales-Granado, M. Santamaria, O. Neth, O. Ekwall, O. Brodszki, H. Hague, L. A. Devlin, P. Brogan, P. D. Arkwright, A. Riordan, L. McCann, E. McDermott, S. N. Faust, E. Carne

**Affiliations:** 1 Department of Immunology, Royal Free London NHS Foundation Trust, London, UK; 2 Pediatric Immuno-Haematology and Rheumatology Unit, Necker Enfants Malades University Hospital, Assistance Publique-Hôpitaux de Paris (AP-HP), Paris, France; 3 French National Reference Center for Primary Immune Deficiencies (CEREDIH), Necker Enfants Malades University Hospital, Assistance Publique-Hôpitaux de Paris (AP-HP), Paris, France; 4 Fondazione IRCCS Policlinico San Matteo, Centro Per Lo Studio E La Cura Delle Amiloidosi Sistemiche, Pavia, Italy; 5 Department of Pediatric Rheumatology, National Reference Centre for Auto-Inflammatory Diseases and Amyloidosis of Inflammatory Origin (CEREMAIA), Bicêtre hospital, Assistance Publique-Hôpitaux de Paris (AP-HP), Le Kremlin Bicêtre, France; 6 Division of Medicine, National Amyloidosis Centre, University College London, London, UK; 7 Paediatric and Adult Rheumatology, Leeds General Infirmary and Chapel Allerton Hospital, Leeds, UK; 8 Department for Allergology, Rheumatology and Clinical Immunology, University Children’s Hospital Ljubljana, Ljubljana, Slovenia; 9 Department of Clinical Immunology and Internal Medicine, Tertiary Center for Primary Immunodeficiency, National Reference Center for Systemic Autoimmune Diseases (CNR RESO), Hôpitaux Universitaires de Strasbourg, 67000 Strasbourg, France; 10 Université de Strasbourg, INSERM UMR - S1109, 67000 Strasbourg, France; 11 Department of Internal Medicine, Division of Clinical Immunology, Erasmus University Medical Center, Rotterdam, the Netherlands; 12 Department of Immunology, Erasmus University Medical Center, Rotterdam, the Netherlands; 13 Rare Autoinflammatory Conditions Community – UK (RACC – UK), Oxford, UK; 14 http://www.raccuk.com; 15 International Patient Organisation for Primary Immunodeficiencies (IPOPI), Brussels, Belgium; 16 Barcelona PID Foundation, Barcelona, Spain; 17 Paediatric Haematopoietic Stem Cell Transplant Unit, Great North Children’s Hospital (GNCH), Royal Victoria Infirmary, Queen Victoria Road, Newcastle upon Tyne, NE1 4LP UK; 18 Translational and Clinical Research Institute, Faculty of Medical Sciences, Newcastle University, Newcastle upon Tyne, NE2 4HH UK; 19 Irish Vasculitis Organisation, Dublin, Ireland; 20 Center for Chronic Immunodeficiency, Medical Center, Faculty of Medicine, University of Freiburg, University of Freiburg, Freiburg, Germany; 21 Department of Rheumatology and Clinical Immunology, Division of Immunodeficiency, Medical Center, Faculty of Medicine, University of Freiburg, Freiburg, Germany; 22 Department of Pediatric Rheumatology, Pediatric Immune Dysfunction Disease Study Group (GEMDIP), Institut de Recerca Sant Joan de Déu, Sant Joan de Déu Hospital, Barcelona, Spain; 23 Pediatrics Clinic, University of Brescia, ASST Spedali Civili Di Brescia, Brescia, Italy; 24 Department of Paediatric Rheumatology, Leeds Children’s Hospital, Leeds Teaching Hospitals Trust, Leeds, UK; 25 Department of Pediatrics, Institute of Clinical Sciences, The Sahlgrenska Academy, University of Gothenburg, Gothenburg, Sweden; 26 Department of Pediatrics, Queen Silvia Children’s Hospital, Sahlgrenska University Hospital, Gothenburg, Sweden; 27 Pediatric Infectious Diseases and Immunodeficiencies Unit, Hospital Universitari Vall d’Hebron, Universitat Autonoma de Barcelona, Bellaterra, Spain; 28 Jeffrey Modell Diagnostic and Research Center for Primary Immunodeficiencies, Barcelona, Catalonia Spain; 29 University College London Institute of Immunity and Transplantation, London, UK

**Keywords:** Transition, Primary immunodeficiencies, Autoinflammatory diseases, Network

## Abstract

**Background:**

Due to the absence of curative treatments for inborn errors of immunity (IEI), children born with IEI require long-term follow-up for disease manifestations and related complications that occur over the lifespan. Effective transition from pediatric to adult services is known to significantly improve adherence to treatment and long-term outcomes. It is currently not known what transition services are available for young people with IEI in Europe.

**Objective:**

To understand the prevalence and practice of transition services in Europe for young people with IEI, encompassing both primary immunodeficiencies (PID) and systemic autoinflammatory disorders (AID).

**Methods:**

A survey was generated by the European Reference Network on immunodeficiency, autoinflammatory, and autoimmune diseases Transition Working Group and electronically circulated, through professional networks, to pediatric centers across Europe looking after children with IEI.

**Results:**

Seventy-six responses were received from 52 centers, in 45 cities across 17 different countries. All services transitioned patients to adult services, mainly to specialist PID or AID centers, typically transferring up to ten patients to adult care each year. The transition process started at a median age of 16–18 years with transfer to the adult center occurring at a median age of 18–20 years. 75% of PID and 68% of AID centers held at least one joint appointment with pediatric and adult services prior to the transfer of care. Approximately 75% of PID and AID services reported having a defined transition process, but few centers reported national disease-specific transition guidelines to refer to.

**Conclusions:**

Transition services for children with IEI in Europe are available in many countries but lack standardized guidelines to promote best practice.

**Supplementary Information:**

The online version contains supplementary material available at 10.1007/s10875-022-01345-y.

## Introduction

Inborn errors of immunity (IEI) are a heterogeneous group of rare disorders with increased, poor, or absent function in components of the innate and/or adaptive immune systems. Primary immunodeficiencies (PID) and autoinflammatory diseases (AID) represent the majority of known IEI [1, 2] PID are characterized by infection susceptibility, often associated with autoimmune, inflammatory, and malignant complications. In contrast, the dominant feature in AID is recurrent or chronic inflammatory episodes that are systemic, distinct from autoimmune diseases and associated with an increasingly wide clinical phenotype [3, 4]. The majority of AID and more severe forms of PID manifest in childhood although additional manifestations and new complications occur over life. Marked improvements in the diagnosis and management of PID and AID in the last decades have improved the outlook for many patients, but at the same time bring new challenges to the care of these patients—many of whom have multi-morbidity [5–7]. This modification of the natural history of the disease is associated with an increased survival of pediatric patients with PID and AID, who now need transfer to adult services for life-long follow-up [8] Therefore, a well-established interdisciplinary transition protocol is considered a standard of care for these patients and their families’ needs to be carefully planned and managed [9].

Transition has been defined as “A purposeful, planned process that addresses the medical, psychosocial, and educational/vocational needs of adolescents and young adults with chronic physical and medical conditions as they move from child-centered to adult-oriented health care systems”[10], while transition can often be used to refer to the physical move of care to adult services. It is important to make a distinction between this “transfer” and “transition” which also includes the psychological shift whereby the young adult becomes responsible for their own care. To date, despite there already being well-established transition guidelines for patients with other life-long immune-mediated conditions that emerge in childhood, such as rheumatic diseases [11], asthma, and allergy [12], transition pathways for PID and AID have been less clearly defined. Moreover, there are significant differences among European countries regarding pediatric and adult specialist healthcare provision for patients with PID and AID [13], and to date there are no available data for dedicated transition programs for these rare disorders.

The European Reference Networks (ERN) are virtual networks involving healthcare providers across Europe (https://ec.europa.eu/health/ern_en). ERNs aim to tackle complex or rare diseases and conditions that require highly specialized treatment and a concentration of knowledge and resources. To date, there are 24 ERNs involving 25 European countries, over 300 hospitals with over 900 healthcare units and covering all major disease groups. Among them, the European Reference Network on Rare Immunodeficiency, Autoinflammatory and Autoimmune Disease (ERN-RITA) brings together leading European centers with expertise in diagnosis and treatment of rare immunological disorders including PID, AID and autoimmune diseases.

The ERN-RITA Transition Working Group aimed to define the current practices in transition for patients with PID and AID diseases across European health centers as a first step to creating global guidance for transition of these patients.

## Methods

A survey (Supplementary file2) asking about current transition numbers and practice was developed by the ERN-RITA steering group. The survey was circulated to clinicians working in European pediatric PID and AID services in June 2018 through personal and professional networks. To enhance the response rate and capture additional centers, the same questionnaire was sent or re-sent in November 2019 to centers who had not submitted responses to the first circulation. Clinicians were asked to complete separate questionnaires for their cohorts of patients with PID and AID and only one response per center for each was permitted. Ethical approval was not required for collection of this information.

## Results

### Respondent demographics

The survey was sent to 154 clinicians, from 106 centers, in 87 cities in 23 countries in Europe (Supplementary Fig. 1). In total, 76 responses were received from 52 centers, in 45 cities across different 17 countries. Forty-four responses were received from PID centers based in 39 cities in 15 countries, while the remaining 32 responses were obtained from AID centers across 29 cities in 13 countries in Europe. Of the 76 responding centers, 38 centers (50%) are members of the RITA-ERN network.

PID services reported treating larger numbers of patients than AID services (Table 1), reflecting the relative prevalence of the two conditions. Forty-one percent (13/32) of AID services reported treating less than 50 pediatric patients compared with 19% (8/43) of PID services. In contrast, 42% (18/43) of PID services follow more than 200 pediatric patients while only 6% (2/32) of AID centers follow cohorts of this size.

**Table 1:** Demographics of responding centres

Specialty	PID	AI
No. of Countries Responded	15	13
No. patients treated in service		
- < 50 -50–100 -100–200 -200–500 - > 500 -Unknown	8 8 8 13 5 1	13 9 8 2 0 0
No. transition aged patients treated in service		
- < 50 -50–100 -100–200 - > 200 -Unknown	22 13 3 5 1	22 9 0 0 0
No patients transferred to adult services each year		
- < 5 -5–10 -10–20 -20–40 -Unknown	17 15 8 2 2	17 12 2 0 1

As guidance for other immune-mediated conditions recommend that the transition process is started in early adolescence [11, 12] the centers were asked how many pediatric patients with PID or AID they cared for between the ages of 12 and 18. The majority of centers follow fewer than 50 patients in this age group (71%, 22/31 of the AID services and 50%, 22/44 of the PID services). None of the AID services and only 18% (8/44) of the PID services cared for more than 100 patients in this transition age range (Table 1). The vast majority of centers transfer only up to ten patients to adult care each year (91%, 29/32 AID services, and 73%, 32/44 PID services) while a small proportion of PID centers (5%, 2/44) transition 20–40 patients per year (Table 1).

### Logistics of the transition process

**Fig. 1:**
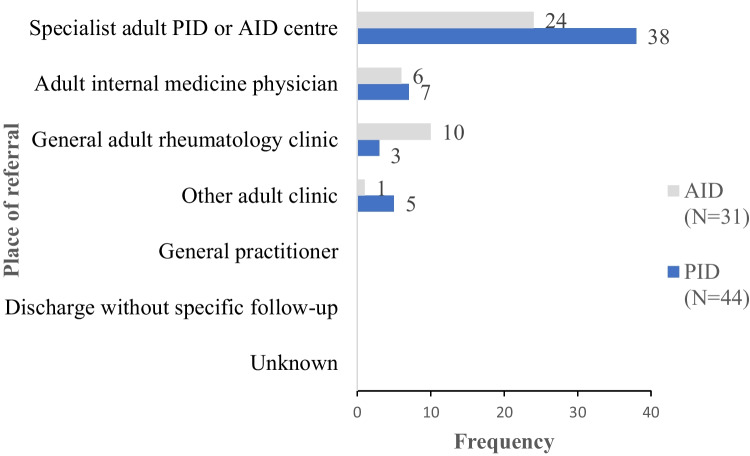
Site of referrals made for adult care

To determine whether patients continue to receive specialist care in adulthood, centers were asked where they transitioned patients to. Fifteen respondents (20%) had more than one option for transfer of care. The vast majority of centers that responded reported referring patients to specialist adult PID/AID centers (86%, 38/44 PID services and 77%, 24/31 AID services: Fig. 1). In keeping with the fact that AID are often cared for within rheumatology services rather than immunology clinics, 32% (10/31) pediatric AID centers referred to adult rheumatologists. No centers discharged without hospital follow-up although a small number referred patients to non-specialist adult internal medicine physicians (16%, 7/44 PID services and 19%, 6/31 AID services).

**Fig. 2:**
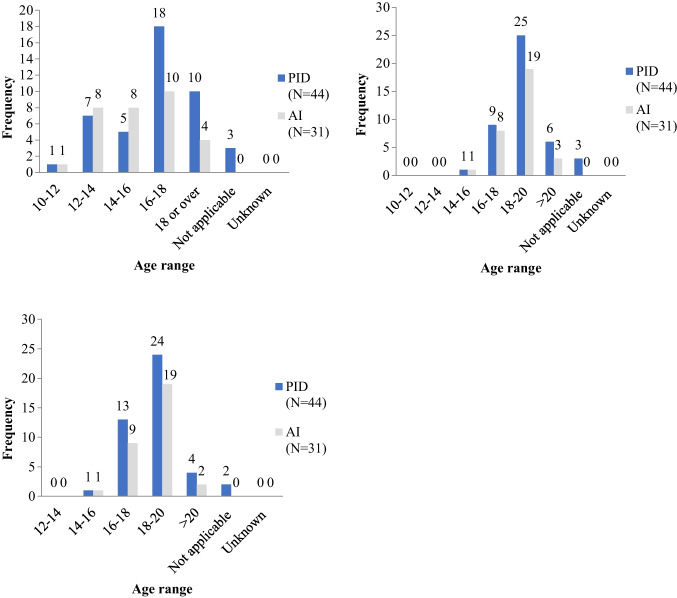
Median age at the start (**a**) and end (**b**) of the transition process and at transfer to an adult center (**c**)

The transition process started at a median age of 16–18 years for both PID and AID services (Fig. 2a). A higher proportion of AID than PID services started transition before the age of 16 years (55%, 17/31 of AID centers compared with 30%, 13/44 of PID centers). A significant minority of services reported initiating the transition process after patients turn 18 (23%, 10/44 of PID services and 13%, 4/31 of AID services). The median age for the end of the transition process was 18–20 years for both PID and AID (Fig. 2b) with a small proportion of centers reporting that the end of transition occurred after the age of 20 years (14%, 6/44 of PID centers and 10%, 3/31 of AID centers). In keeping with this, patients were most frequently transferred to adult care between the ages of 18–20 years (Fig. 2c) with a smaller proportion of centers transferring patients before 18 years of age (32%, 14/44 of PID services and 32%, 10/31 of AID services). Delay in transfer to over 20 years of age was reported by a minority of centers (9%, 4/44 of PID centers and 6%, 2/31 of AID centers).

The majority of centers reported that patients were referred to an adult center a similar distance from the patient’s home (75%, 33/44 pediatric PID services and 23/31, 74% pediatric AID services; Supplementary Fig. 2). Only one AID center reported that the adult center was further from the patient’s home but 20%, 9/44 PID services and 19%, 6/31 AID services responded that the distance between the patient’s home and adult center varied for each patient.

### Transition partner and process

The majority of the response sites reported having formal transition partners in place (91%, 40/44 of PID services and 94%, 29/31 AID services). Transfer was typically directly to adult centers, with only 7% (3/44) PID services and 16% (5/31) AID services transitioning to intermediate adolescent services (Table 2). Few PID or AID respondents reported transferring their patients to adult care services with dedicated young adult clinics (11%, 5/44 PID and 29%, 9/31 AID services). Joint appointments with healthcare professionals from pediatric and adult services were common prior to the transfer of care (75%, 33/44 PID and 68%, 21/31 AID centers), involving physicians, nurses, psychologists, and specialists from other medical teams. The number of joint clinics held for each patient was determined by the severity of illness. PID services described holding 1–5 joint clinics, with 4–5 appointments for patients with more complex diseases. In contrast, the AID services reported holding 1–3 appointments for each patient. The vast majority of the services examined described full integration of patient records with the adult care provider, with only 21% (9/43) PID centers and 27% (8/30) AID services responding negatively to this item (Table 2).

**Table 2:** Access to and Use of Transition Resources

	PID	AI
Patients transitioned to adolescent services prior to moving to adult care	3/44 (7%)	5/31 (16%)
Transfer to adult services with designated young adult clinics	5/44 (11%)	9/31 (29%)
Joint transition clinics with paediatric and adult services	33/44 (75%)	21/31 (68%)
Full integration of records across paediatric and adult services	34/43 (79%)	22/30 (73%)
Transition-specific research programs in the paediatric service	9/44 (20%)	9/31 (29%)

**Fig. 3:**
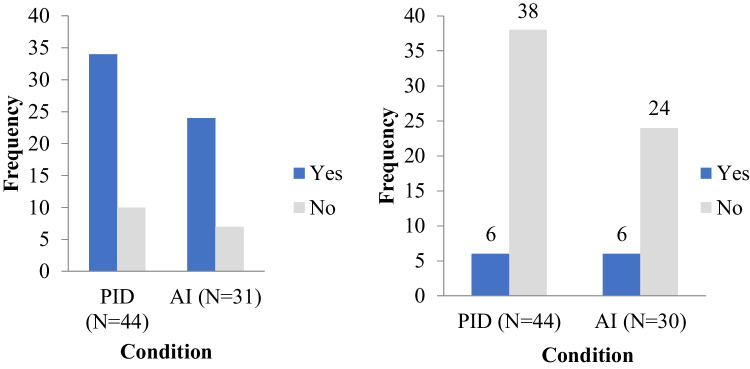
Use of defined transition processes (**a**) and access to national disease-specific guidelines for transition (**b**)

Just over three quarters of PID and AID services reported having a defined transition processes for transfer of care to adult services (34/44 and 24/31 respectively; Fig. 3a). However, the majority of PID and AID services reported a lack of national disease-specific transition guidelines to refer to (86%, 38/44 and 80%, 24/30 respectively; Fig. 3b). The PID services describing the complete or partial use of national transition guidelines were located in Poland and the UK, while the AID services were based in Italy, Finland, and the UK. Few centers reported transition-specific research programs at their site (20%, 9/44 PID and 29%, 9/31 AID centers; Table 2).

The majority of centers did not report specific difficulties in identifying adult care for their patients (Supplementary Fig. 3). However, for those who did, the lack of specialist adult center (9%, 8/23 PID and 27%, 3/11 AID centers) was the biggest difficulty. If reporting problems, the majority of clinicians identified a number of different weaknesses of the transition process in their services—with 54 respondents (71%) listing more than one difficulty. Lack of time to prepare the documents and/or funding and resource in adult services, fragmentation of adult services, lack of holistic care in both and adult pediatric services, lack of suitable centers to transition to, and patients not wanting to engage were reported as the main weaknesses of the transition process (45%, 33%, 33%, 26%, 22%, 22%, and 18% of services, respectively) (Supplementary Table 1). This survey did not capture specific problems with the suitability of adult services for transition. However, responders did note that it is often the transfer of services for all the various comorbidities in the adult sector, rather than transfer for PID itself that is problematic, and that different arrangements for transition may be required for different AID diagnoses. The lack of provision for patients with learning disability or mental health difficulties compared to pediatrics was also noted as a weakness of the process.

Prior to transfer, services report discussing a number of different topics or concerns with their patients, with the majority of services discussing the patient’s understanding of the disease (92% of services), and medication (88%), genetics (86%), compliance with treatment (88%), and responsibility for own health care (82%), preference for transition center (71%) and expectations of adult services (70%) (Supplementary Table 2). Around half of services discussed mental health or wellbeing, vocational expectations, sexual health, substance use, and/or life expectancy.

### Challenges for smaller centers

The data were reviewed to determine whether there were differences between sites transferring more or less than 10 patients to adult services each year. Sixty-one services transferred less than 10 patients each year, but only 12 transferred more than 10. Services transitioning less than ten patients each year treated fewer pediatric patients in total and within the 12–18 year age range (Supplemental Fig. 4a and b). A higher percentage of centers transferring more than ten patients a year reported transferring patients between the ages of 16 and 18, having a defined process for transition, having integrated medical files, and having joint clinics with adult and pediatric centers (Table 3). A higher percentage of centers transferring less than ten patients a year reported initiating transition and/or transferring care after the age of 18, transferring to services with a specialist young adult clinic, and having disease-specific guidelines (Table 3).

**Table 3:** Differences between services transferring more and less than 10 patients a year

	< 10	> 10
Initiate transition before 16 years	25/60 (42%)	4/12 (33%)
Initiate transition between 16–18 years	22/60 (37%)	6/12 (50%)
Initiate transition after 18 years	12/60 (20%)	1/12 (8%)
Transfer between 14–16 years	2/60 (3%)	0/12 (0%)
Transfer between 16–18 years	16/60 (27%)	5/12 (42%)
Transfer between 18–20 years	36/60 (60%)	6/12 (50%)
Have formal transition partner	57/60 (95%)	11/12 (92%)
Defined process of transition	47/60 (78%)	11/12 (92%)
Disease specific transition guidelines	10/59 (17%)	1/12 (8%)
Integration of medical files	45/59 (76%)	10/11 (91%)
Joint transition clinics with paediatric and adult services	44/60 (73%)	10/12 (83%)
Patients transitioned to adolescent services prior to moving to adult care	6/60 (10%)	2/12 (17%)
Transfer to adult services with designated young adult clinics	12/60 (20%)	1/12 (8%)
Transition-specific research programs in the paediatric service	14/60 (23%)	3/12 (25%)

## Discussion

Successful transition from pediatric to adult services is a key component of clinical care for chronic conditions that predicts treatment adherence and medical outcome [14–17]. Transition for rare diseases (RD), such as PID and AID, present specific challenges, particularly for patients with multisystem disorders. In general, across Europe, pediatric PID and AID care occurs mainly in specialist centers of varying sizes while dedicated adult services are less well defined. Thus, provision for patients with PID and AID exhibits regional differences, creating variation in the patient care pathway as seen for other RD—a specific issue that ERNs seek to address [18]. This ERN-RITA study is the first report of transition practices for patients with PID and AID across European health centers and aimed to understand the current processes and key barriers to transition for these RD.

This survey was circulated to pediatric centers caring for patients with PID and AID and therefore the results reflect the pediatric heath care professional (HCP) perspective. Our results report that the majority of pediatric centers have transition processes in place although only a minority start early in adolescence, which is recommended as it leads to better knowledge and skills and long-term outcome [19]. As the majority of the pediatric PID and AID services begin the transition process when the patient is between the ages of 16 and 18, the processes of transition and transfer to adult services often occur closely together, reducing time for adjustment for patients and their families. The most frequently reported factor influencing age of transfer was “patient considered ready for transition,” but data were not gathered on whether readiness for transition is evaluated by patients themselves or by medical teams. This is especially important because 18% of the total respondents reported patient unwillingness to engage as one of the main weaknesses of their transition program. Furthermore, patient readiness has been observed to influence optimal transition in terms of health-related outcomes and the development of self-management skills by patients [20, 21]. Generic transition guidelines suggest that the process of transition should start when the patient is 13 or 14 at the latest [22], in order to enhance patient familiarity with the adult unit and staff prior to transfer and augment patient readiness for transfer. It may be advisable for centers to administer questionnaires measuring the patient’s readiness to transition and tailor transition plans accordingly to address the unique concerns of each patient.

Meeting the adult team prior to transfer has been identified as a significant factor in predicting better outcomes for young people living with long-term conditions [23]. This occurred in the majority of, but not all, centers transitioning young adults with PID or AID through the provision of joint in-person or virtual clinics attended by HCPs from both pediatric and adult centers prior to the transfer of care. Although special care services for young people are recommended by the International Patient Organisation for Primary immunodeficiencies (IPOPI) [24], only a small proportion of PID and AID clinics surveyed reported transferring their patients to adult services with designated young adult clinics, with even fewer transferring their patient cohort to adolescent immunology clinics prior to adult services. These practices may reduce the confidence of young adults to manage their health, as only 60% or less of all the services in this sample discuss broader psychosocial and occupational concerns like fertility, sexual health, substance use, mental health and wellbeing, vocational and life expectations, and health insurance with their patients prior to transfer (see Supplementary Table 3). Transition to adolescent centers or dedicated young adult clinics may thus allow for the provision of holistic care to young patients by addressing health and wellbeing concerns specific to this developmental stage alongside those stemming from their IEI.

While a minority of services reported difficulties in transferring patients to adult services, there were shared difficulties in the process of transition where these were reported, particularly the lack of engaged specialist adult services, which may be a contributing factor to delayed transition in some centers. Fragmentation of healthcare teams across different services also may present challenges in identifying suitable adult centers equipped to offer all required aspects of interdisciplinary care for young patients with multimorbidity. In addition, many services identified lack of time to prepare documentation as the main weakness of their transition program, and/or incomplete integration of medical records across pediatric and adult services. It is our opinion that each pediatric service, and adult services who receive transfer should have a nominated transition lead and dedicated administrative staff to oversee documentation and allow for more seamless transition.

At present, while the majority of the pediatric PID and AID clinics across Europe reported using a defined transition process to transfer patients to adult services, few reported having access to national disease-specific guidelines for transition. In the interest of optimizing health-related outcomes for adolescents undertaking increased responsibility of their health, it may be advantageous to develop disease-specific guidelines that outline gold-standard transition practices and outcomes based on consensus from multidisciplinary healthcare professionals. Since the completion of this study, the French Network for Autoimmune and Autoinflammatory Diseases and Italian Primary Immunodeficiency Network have published their consensus statements on management of transition from pediatric to adult care in patients affected with childhood-onset IEI aimed at improving clinical practice in this area, with a helpful focus on different requirements for specific types of IEI [25, 26]. Future work could also focus on guidelines to support the clinical management of IEI patients with certain comorbidities (such as learning disabilities) and those requiring long-term follow-up after corrective or novel treatments (such as stem cell therapies).

This study was the first large-scale survey of transition practices for primary immunodeficiency and autoinflammatory conditions in Europe. However, it is important to recognize that there may be a bias in reporting related to the geography of the responding centers. The majority of the response sites in this study were based in the UK, Belgium, Italy, and Spain, and these findings may not be representative of transition programs and patient cohorts in regions with fewer response sites, like Central and Eastern Europe and Scandinavia. The challenges are likely to be different in different regions which may have an impact for translating guidelines to practice. In addition, most information came from countries with established Adult Immunology Services. This study did not examine a patient and family/carer perspective, which may be different to a professional overview. Hence, it will be important for future research to explore patient and family/carer experiences of transition and their reported difficulties with the process. Finally, it is possible that findings from this study were affected by selection bias—with centers already invested in transition practices being more likely to respond to the survey. Hence, results from this survey may not reflect transition policies in centers with less-established transition programs.

Further work is needed to develop comprehensive national and international illness-specific transition guidelines for PID and AI. These should establish practices to enable young people to develop independence with respect to their healthcare and address aspects of life and wellbeing impacted by specific health conditions. Future work should also consider factors conducive to successful and unsuccessful transition, for example, avoiding transfer of care during active disease phase, and any guidelines should highlight the need to understand the proportion of young patients who are lost to follow-up or show a decline in treatment adherence subsequent to the transfer of care. Accordingly, the next step for the RITA–ERN transition group is to develop good practice recommendations for transition in these populations and identify the outcome measures that can be used in future studies to assess the impact of transition guidance on the long-term health of adults living with childhood-onset IEI.

## Supplementary Information

Below is the link to the electronic supplementary material.

Supplementary file1 (DOCX 209 KB)

Supplementary file2 (PDF 251 KB)

Supplementary file3 (DOCX 19.1 KB)

## Data Availability

The datasets generated during and/or analyzed during the current study are available from the corresponding author on reasonable request.
