# Supplementary material for: Current Transition Practice for Primary Immunodeficiencies and Autoinflammatory Diseases in Europe: a RITA-ERN Survey
Source: J Clin Immunol. 2022 Oct 12;43(1):206–16. doi: 10.1007/s10875-022-01345-y (PMC9840587; doi:10.1007/s10875-022-01345-y)
Supplement: Supplementary file 1 — Supplementary file1 (DOCX 209 KB) [file 10875_2022_1345_MOESM1_ESM.docx]

Supplementary Figure 1: Responding centres


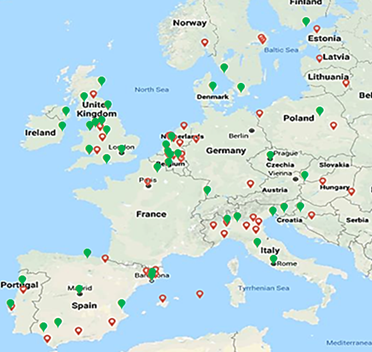


Contacted sites Response sites

Supplementary Figure 2: Distance to adult care service.

Supplementary Figure 3: Difficulties in identifying adult centre for transfer of care

Supplementary Figure 4: (a)Number of paediatric patients treated at centres transferring more or less than 10 patients per year (b) and number of patients aged twelve to eighteen treated at centres transferring more or less than 10 patients per year.
