# Supplementary material for: Current Transition Practice for Primary Immunodeficiencies and Autoinflammatory Diseases in Europe: a RITA-ERN Survey
Source: J Clin Immunol. 2022 Oct 12;43(1):206–16. doi: 10.1007/s10875-022-01345-y (PMC9840587; doi:10.1007/s10875-022-01345-y)
Supplement: Supplementary file 2 — Supplementary file2 (PDF 251 KB) [file 10875_2022_1345_MOESM2_ESM.pdf]

Transition within Immunology Services

Please answer every question as best as possible. The questionnaire needs to be completed and submitted in one sitting. Thank you.

1. **Email address** *

Centre Details

2. **Hospital:**

3. **City:**

4. **Country:**

5. **Lead clinician:**

6. **Referral base (tick all that apply):**
Check all that apply.

- [ ] Local
- [ ] Regional
- [ ] National
- [ ] Unknown
- [ ] Other:

7. **Are you completing this for: (If both, please fill in a separate questionnaire for each, and indicate your response for the current questionnaire. To access an additional copy of the form, click the link in the original email for a second time once you have submitted the current one.)**
Mark only one oval.

- ( ) Primary Immunodeficiency Disease
- ( ) Autoinflammatory Disorders

8. **What number of paediatric patients with this condition are cared for at your centre?**
Mark only one oval.

- <50
- 50-100
- 100-200
- 200-500
- >500
- Unknown

9. **What number of patients aged 12-18 with this condition are cared for at your centre?**
Mark only one oval.

- <50
- 50-100
- >200
- Unknown

10. **What number of patients do you transfer to adult services each year?**
Mark only one oval.

- <5
- 5-10
- 10-20
- 20-40
- >40
- Unknown

Transition Process

11. **Where do you refer paediatric patients for adult care?**
Check all that apply.

- [ ] Specialist adult PID/autoinflammatory centre
- [ ] General adult rheumatology clinic
- [ ] Adult internal medicine physician
- [ ] General practitioner
- [ ] Discharge without specific follow up
- [ ] Other adult clinic
- [ ] Unknown

12. **Do you have formal transition partner(s)?**
Mark only one oval.

- Yes
- No

13. **What proportion of your patients transition to your formal transition partner?**
Mark only one oval.

- ◯ >75%
- ◯ 50-75%
- ◯ 25-50%
- ◯ <25%
- ◯ Unknown
- ◯ Not applicable

14. **Do you have a defined process for transition?**
Mark only one oval.

- ◯ Yes
- ◯ No

15. **Do you have national disease-specific guidelines for transition?**
Mark only one oval.

- ◯ Yes
- ◯ No

16. **Do you follow these at your centre?**
Mark only one oval.

- ◯ Yes
- ◯ No
- ◯ Partially

17. **Does your transition program include patients who have had Hematopoietic Stem Cell Transplantation in childhood?**
Mark only one oval.

- ◯ Yes
- ◯ No
- ◯ Not yet (future aspiration)

18. **Who chooses the adult centre? (tick all that apply, eg where decision is a consensus)**
Check all that apply.

- ☐ The medical team
- ☐ The parents
- ☐ The patient
- ☐ No choice: there is only one centre available
- ☐ Funder (eg health insurance)
- ☐ Unknown
- ☐ Not applicable

19. **What influences your choice of adult centre? (tick all that apply)**
Check all that apply.

- [ ] Expertise of the adult team
- [ ] Other services available at that centre
- [ ] Local location for the patient
- [ ] Professional connection with the adult team
- [ ] Funding options
- [ ] Unknown
- [ ] Not applicable

20. **Compared to the paediatric centre, is the adult centre usually:**
Mark only one oval.

- ( ) Further from the patient's home
- ( ) Similar distance from the patient's home
- ( ) Nearer to the patient's home
- ( ) Variable for each patient
- ( ) Unknown
- ( ) Not applicable

21. **Do you have difficulty identifying a specialist centre for adult care?**
Mark only one oval.

- ( ) Yes
- ( ) No

22. **If yes, what are the reasons? (tick all that apply)**
Check all that apply.

- [ ] Lack of specialist adult centres
- [ ] Lack of engagement from existing/specialist adult services
- [ ] Distance for the patient to travel to a specialist centre
- [ ] Lack of funding for follow up of adult patients
- [ ] Unknown
- [ ] Not applicable
- [ ] Other: ____________________

If a transition program is in place:

23. **What median age does this start at?**
Mark only one oval.

- 10-12
- 12-14
- 14-16
- 16-18
- 18 or over
- Unknown
- Not applicable

24. **What median age does this end at?**
Mark only one oval.

- 10-12
- 12-14
- 14-16
- 16-18
- 18-20
- >20
- Unknown
- Not applicable

25. **At what age do you transfer to the adult centre?**
Mark only one oval.

- 12-14
- 14-16
- 16-18
- 18-20
- >20
- Unknown
- Not applicable

26. **What material/program do you use?**

27. **What factors influence age of transfer? (tick all that apply)**
Check all that apply.

- [ ] End of transition program
- [ ] Patient considered to be ready for transition
- [ ] Medically stable
- [ ] Age of the patient
- [ ] Paediatric centre can no longer offer care
- [ ] Unknown
- [ ] Not applicable
- [ ] Other: ____________

28. **Are patients transitioned to intermediate adolescent services prior to moving to the adult service?**
Mark only one oval.

- ◯ Yes
- ◯ No

29. **Do you have joint clinics with the adult team prior to transfer to adult care?**
Mark only one oval.

- ◯ Yes
- ◯ No

30. **If yes, how many joint appointments occur per patient?**

____

31. **Do you have the ability for virtual joint clinics between paediatric and adult teams?**
Mark only one oval.

- ◯ Yes
- ◯ No

32. **If yes, please give further details:**

____
____
____
____
____

33. **Does the adult centre have a designated young adult clinic?**
Mark only one oval.

- ◯ Yes
- ◯ No

34. **What professionals are involved in the transition process? (tick all that apply)**
Check all that apply.

- [ ] Paediatric physician
- [ ] Adult physician
- [ ] Nurse
- [ ] Psychologist
- [ ] Youth Worker
- [ ] Social Worker
- [ ] Pharmacist
- [ ] Specialists from other medical teams
- [ ] Unknown
- [ ] Not applicable
- [ ] Other

35. **If 'specialists from other medical teams' or 'other', please specify here:**

36. **What professionals are involved in the transfer appointment? (tick all that apply)**
Check all that apply.

- [ ] Paediatric physician
- [ ] Adult physician
- [ ] Nurse
- [ ] Psychologist
- [ ] Youth worker
- [ ] Social worker
- [ ] Pharmacist
- [ ] Specialists from other medical teams
- [ ] Other
- [ ] Unknown
- [ ] Not applicable

37. **If 'specialists from other medical teams' or 'other', please specify here:**

38. **What information is transitioned to the adult team? (tick all that apply)**
Check all that apply.

- [ ] Transition letter
- [ ] Full medical history including co-morbidities
- [ ] Imaging files
- [ ] Relevant test results (eg blood/lung function)
- [ ] Vaccination history
- [ ] Details of conditioning and donor if HSCT
- [ ] Genetic report
- [ ] Family pedigree
- [ ] Psychological well-being/mental health
- [ ] Learning disability/educational needs
- [ ] Education/occupation plan
- [ ] Social work involvement
- [ ] Medication details
- [ ] Specific issues about procedure fears (eg needlephobia)
- [ ] Unknown
- [ ] Not applicable
- [ ] Other:

39. **Is there full integration of records between paediatric and adult services (eg electronic case record)?**
Mark only one oval.

- ( ) Yes
- ( ) No

40. **Do you have dedicated administrative time (e.g. a secretary) to prepare transition documents?**
Mark only one oval.

- ( ) Yes
- ( ) No

41. **Prior to transfer do you specifically discuss the following with the patient? (tick all that apply)**
Check all that apply.

- [ ] Understanding of disease
- [ ] Genetic basis and heritability
- [ ] Fertility and parenthood (if previous HSCT)
- [ ] Contraception and sexual health
- [ ] Substance use (alcohol/drugs)
- [ ] Understanding of current medications
- [ ] Compliance with current treatment regimen
- [ ] Preference for transition centre
- [ ] Expectations of adult services
- [ ] Patient responsibility for own health care
- [ ] Mental health and well-being
- [ ] Vocational expectations (work and education planning)
- [ ] Life expectancy
- [ ] Health insurance
- [ ] Unknown
- [ ] Not applicable

42. **What, in your opinion, are the main weaknesses of your transition program? (tick all that apply)**
Check all that apply.

- [ ] Lack of suitable centres to transition to
- [ ] My patients do not want to engage
- [ ] I do not have engagement from the paediatric team
- [ ] I do not have engagement from the adult team
- [ ] I do not have time to prepare the documentation
- [ ] Medication that the patient is receiving is not available in the adult centre
- [ ] Lack of resource and funding for transition within the adult service
- [ ] Fragmentation of services in adult centres
- [ ] Lack of holistic care at the paediatric centre (eg psychology)
- [ ] Lack of holistic care at the adult centre (eg psychology)
- [ ] Unknown
- [ ] Not applicable
- [ ] Other: ____________________

43. **Do you have keep a database of transitioned patients?**
Mark only one oval.

- ( ) Yes
- ( ) No

44. **Do you have any transition-specific research program in your department?**
Mark only one oval.

- Yes
- No

Powered by
Google Forms
