# Supplementary material for: Current Transition Practice for Primary Immunodeficiencies and Autoinflammatory Diseases in Europe: a RITA-ERN Survey
Source: J Clin Immunol. 2022 Oct 12;43(1):206–16. doi: 10.1007/s10875-022-01345-y (PMC9840587; doi:10.1007/s10875-022-01345-y)
Supplement: Supplementary file 3 — Supplementary file3 (DOCX 19.1 KB) [file 10875_2022_1345_MOESM3_ESM.docx]

Supplementary Table 1: Weaknesses of the transition programme (N = 76)

| Weaknesses of Transition Program | Number of responses | % of respondents selecting each option |
| --- | --- | --- |
| I do not have time to prepare the documentation | 34 | 45% |
| Lack of resource and funding for transition within the adult service | 25 | 33% |
| Fragmentation of services in adult centres | 25 | 33% |
| Lack of holistic care at the adult centre (e.g. psychology) | 20 | 26% |
| Lack of holistic care at the paediatric centre (e.g. psychology) | 17 | 22% |
| Lack of suitable centres to transition to | 17 | 22% |
| My patients do not want to engage | 14 | 18% |
| I do not have engagement from the adult team | 6 | 8% |
| Lack of shared clinics between paediatric and adult clinics and lack of dedicated adolescent physician | 1 | 1% |
| Lack of appointed transition nurse | 1 | 1% |
| Forced too early transition due to legislation | 1 | 1% |
| Patients do not want to move to adult services | 1 | 1% |
| Patients feel out of their comfort zone | 1 | 1% |
| Lack of dedicated administrative team (e.g. a secretary) | 1 | 1% |
| Medication that the patient is receiving is not available in the adult centre | 1 | 1% |
| Insufficient funding to provide services (need to use external funds) | 1 | 1% |
| Strong personal connection with caregiver in paediatrics - long transition time | 1 | 1% |
| I do not have engagement from the paediatric team | 1 | 1% |
| Blank/Not Applicable | 7 | 9% |

Supplementary Table 2: Topics Discussed with Patients Prior to Transfer (N = 76)

| **Topics Discussed** | **Number of responses** | **% of respondents selecting each option** |
| --- | --- | --- |
| Understanding of disease | 70 | 92% |
| Understanding of current medications | 67 | 88% |
| Compliance with current treatment regimen | 67 | 88% |
| Genetic basis and heritability | 65 | 86% |
| Patient responsibility for own health care | 62 | 82% |
| Preference for transition centre | 54 | 71% |
| Expectations of adult services | 53 | 70% |
| Fertility and parenthood (if previous HSCT) | 44 | 58% |
| Vocational expectations (work and education planning) | 42 | 55% |
| Contraception and sexual health | 42 | 55% |
| Substance use (alcohol/drugs) | 40 | 53% |
| Mental health and well-being | 39 | 51% |
| Life expectancy | 34 | 45% |
| Health insurance | 10 | 13% |
| Blank/Not applicable | 4 | 5% |
